# Description of a new species of cynopoeciline killifish (Cyprinodontiformes, Aplocheilidae), possibly extinct, from the Atlantic Forest of south-eastern Brazil

**DOI:** 10.3897/zookeys.867.34034

**Published:** 2019-07-29

**Authors:** Wilson J.E.M. Costa

**Affiliations:** 1 Laboratory of Systematics and Evolution of Teleost Fishes, Institute of Biology, Federal University of Rio de Janeiro, Caixa Postal 68049, CEP 21941-971, Rio de Janeiro, Brazil Federal University of Rio de Janeiro Rio de Janeiro Brazil

**Keywords:** Biodiversity, conservation, moist tropical forest, systematics, taxonomy

## Abstract

Specimens found between 1985 and 1988 in the Magé River Basin, south-eastern Brazil were misidentified as *L. splendens*. The recent rediscovery of other specimens in the Estrela River Basin near the type locality of *L. splendens* has clarified the species’ concept, making it possible to recognise the Magé River Basin specimens as a new species. The new species is herein described as *Leptopanchax sanguineus***sp. nov.** and is distinguished from all other cynopoecilines by a unique colour pattern in males, including red bars with sinuous margins. It was collected in a well-preserved, temporary shallow swampy area within dense moist forest, but since 1990 the species has not been found again. *Leptopanchax sanguineus***sp. nov.** is one of three species of cynopoeciline killifishes living in lowland moist forests of the coastal plains of Rio de Janeiro State, where the greatest diversity of endemic cynopoecilines is concentrated. Each of these species has been recorded a single time in the last 30 years, a surprisingly low record attributable to intense deforestation during the last several decades resulting in small fragmented lowland moist forests of today. This study indicates that seasonal killifishes adapted to uniquely live in this kind of habitat should be regarded with special concern in studies evaluating conservation priorities.

## Introduction

The Atlantic Forest of south-eastern Brazil encompasses one of the most species-rich biota in the world, with a high diversity of plants and animals (Myers et al. 2000). Although the greatest part of the original Atlantic Forest was extirpated in the last three centuries and consequently several endemic species became endangered or even extinct, new species are still being recognised in recent years (Tabarelli et al. 2005; Costa 2016a). Aplocheilid killifishes are represented in the Atlantic Forest by 14 genera and over 45 valid species (Costa 2009, 2014, 2016b; Costa and Amorim 2013, 2014; Costa et al. 2014), of which six genera and 18 species belong to the tribe Cynopoecilini (Costa 2008, 2016b; Ferrer et al. 2014). Like several other South American and African aplocheiloids, cynopoecilines are seasonal killifishes, which uniquely have their entire life cycle restricted to temporary pools and swamps formed during rainy seasons (Myers 1942; Costa 2002a, 2009).

The greatest species diversity of cynopoecilines is concentrated in the coastal plains of Rio de Janeiro State, south-eastern Brazil (i.e., eight valid endemic species in three genera, of which two genera are endemic), with most taxa consisting of miniature species not surpassing 25 mm standard length (SL) and exhibiting high diversification of morphological traits (Costa 2008, 2016a). Different kinds of vegetation formations sheltering distinct seasonal killifish habitats are present in this region, including temporary pools in seasonally dry forests and coastal restingas, and seasonal swamps in dense moist forests (Costa 1995, 2009, 2016a). This region also contains the greatest occurrence of cynopoeciline species threatened with extinction in South America, some of them critically endangered or presumably extinct (Costa 2002b, 2009, 2012), with most taxa poorly represented in ichthyological collections and not collected in recent years (Costa 2016a).

Among areas of endemism for seasonal killifishes in the Rio de Janeiro coastal plains is the area encompassing river basins draining the southern flank of the coastal mountain range, Serra do Mar, and flowing into the Baía de Guanabara (i.e. Guanabara Bay area in Costa 2009, 2012; hereafter GBA). Three species of seasonal killifishes have been reported to occur in the GBA: *Leptolebias marmoratus* (Ladiges, 1934), *Leptopanchax opalescens* (Myers, 1942) and *Leptopanchax splendens* (Myers, 1942). This area was formerly occupied by a dense moist forest, but presently, after over 60 years of intense deforestation, the forest is restricted to small enclaves within urban areas. All three species were primarily recorded from the Estrela River Basin, in the western portion of the GBA (Myers 1942). Whereas *L. opalescens* was found in open vegetation habitats close to the forest border, *L. marmoratus* and *L. splendens* were found only within the dense moist forest (Myers 1942; Costa et al. 1988; Costa 2009). All three species were considered extinct after extirpation of known habitats in the 1950s, but *L. marmoratus* and *L. opalescens* were rediscovered some decades after, in neighbouring basins of western GBA (Costa 2002c, 2013). Costa and Lacerda (1988) redescribed *L. splendens* on the basis of old collections deposited in museums. They also provided colour photographs of a male tentatively identified as *L. splendens* and its habitat, consisting of a well-preserved forest area in the Magé River Basin, eastern portion of the GBA. However, the recent rediscovery of a population of *L. splendens* near its type locality in the Rio Estrela basin, over 60 years after its last record, has shown that this species is probably endemic to the Estrela River Basin in the western part of the GBA (Costa et al. 2019) and is not conspecific with specimens from the Magé River Basin in the eastern part of the GBA. Specimens of both populations differ greatly in several morphological characters, including fin ray, scale and vertebra counts, extent and relative position of fins, presence of filamentous rays on unpaired fins, presence of contact organs on male pectoral fin, presence of dermosphenotic bone, and male colour pattern. The new species from the Magé River Basin, which was misidentified as *L. splendens* in the last three decades (Costa and Lacerda 1988; Costa 2002b, 2009), is herein described.

## Material and methods

The description of the new species was based on specimens collected over 30 years ago and then preserved for study, deposited in the ichthyological collections of the Museu Nacional, Universidade Federal do Rio de Janeiro, Rio de Janeiro (MNRJ) and Museu de Zoologia, Universidade de São Paulo, São Paulo (MZUSP). Colouration characters were analysed and described based on photographs taken from a male collected in 1985 (published in Costa and Lacerda 1988: fig.1) and notes taken from direct observation in aquaria of live specimens collected in 1985 and 1987; colouration characters were also checked in photographs of live specimens born in aquaria, published in Costa (1995: fig. 113) and Seegers (2000: fig. S31853-4). Since no new photographs are available, a coloured pencil drawing based on available material and rigorously following fish proportions and colours was made to illustrate the new species. Measurements and counts follow Costa (1988). Measurements are presented as percentages of SL, except for those related to head morphology, which are expressed as percentages of head length. Fin ray counts include all elements. Osteological data were taken from cleared and stained specimens prepared following Taylor and Van Dyke’s (1985) protocol; the abbreviation C&S indicates specimens prepared for osteological observation and preserved in glycerine. Terminology for frontal squamation followed Hoedeman (1958), and for the cephalic neuromast series followed Costa (2001). Comparative material is listed in Costa (2016a) and Costa et al. (2019). The map illustrating species distribution was generated using QGIS Geographic Information System, Open Source Geospatial Foundation Project.

## Results

### Leptopanchax sanguineus sp. nov.

Taxon classificationAnimaliaCyprinodontiformesAplocheilidae

73b1f7db-93c9-5914-8973-30f742847893

http://zoobank.org/C106BC45-F740-432D-9288-881A721BD7EF

**Figure 1.**
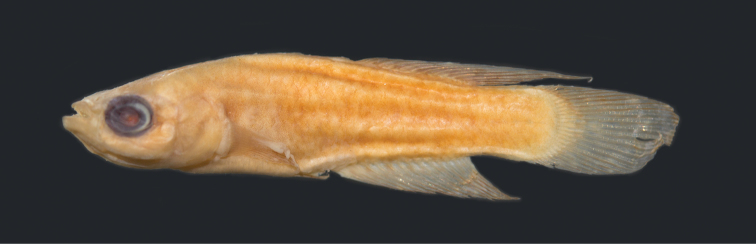
*Leptopanchax sanguineus* sp. nov., MNRJ 51331, holotype, male, 20.9 mm SL. Scale bar: 5 mm.

**Figure 2.**
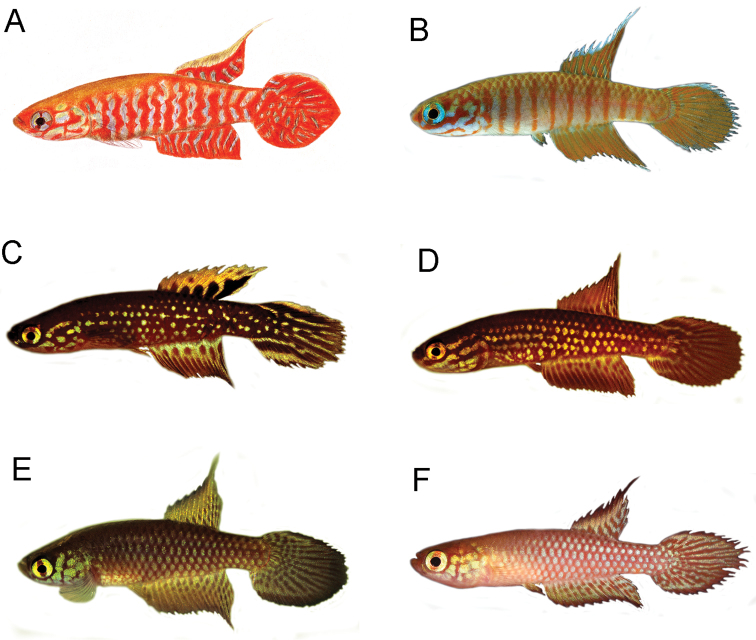
Male fin morphology and life colour patterns in *Leptopanchax*. **A** coloured pencil drawing illustrating *L. sanguineus* sp. nov. in life, about 20 mm SL**B***L. splendens*, UFRJ 6902, 22.7 mm SL**C***L. aureoguttatus*, UFRJ 6331, 22.3 mm SL**D***L. itanhaensis*, UFRJ 6453, 20.7 mm SL**E***L. citrinipinnis*, UFRJ 8899, 20.6 mm SL**F***L. opalescens*, UFRJ 8986, 20.2 mm SL.

Figs 1 , 2A , Table 1

#### Holotype.

MNRJ 51331, male, 20.9 mm SL; Brazil: Rio de Janeiro State: Magé Municipality: temporary swamp within dense moist forest in a private reserve (Reserva Particular do Patrimônio Natural Campo Escoteiro Geraldo Hugo Nunes), Magé River Basin, near the village of Citrolândia, 22°34'57"S, 43°02'08"W, altitude about 30 m above sea level (a.s.l.); M. T. C. Lacerda and K. Tanizaki, August 1987.

#### Paratypes.

MNRJ 11413, 2 males, 20.6–20.8 mm SL; MZUSP 38443, 1 male, about 20 mm SL, 1 female, about 15 mm SL (C&S); collected with holotype.

**Table 1.** Morphometric data of *Leptopanchax sanguineus* sp. nov.

	**Holotype**	**Paratypes**	
	**male**	**male**	**male**
Standard length (mm)	20.9	20.8	20.6
% **of standard length**			
Body depth	25.9	27.4	26.2
Caudal peduncle depth	14.9	16.1	15.6
Pre-dorsal length	61.0	61.8	59.7
Pre-pelvic length	52.4	53.1	50.6
Length of dorsal-fin base	25.7	25.1	26.4
Length of anal-fin base	31.4	29.0	31.4
Caudal-fin length	35.3	-*	34.7
Pectoral-fin length	22.3	22.5	21.2
Pelvic-fin length	9.1	9.4	8.9
Head length	29.6	29.8	31.1
% **of head length**			
Head depth	76.7	77.4	77.4
Head width	71.0	75.2	69.5
Snout length	10.2	11.1	10.9
Lower jaw length	20.9	21.6	17.3
Eye diameter	36.1	36.5	33.8

#### Diagnosis.

*Leptopanchax sanguineus* differs from other cynopoecilines, except *L. splendens*, by the presence of red bars on the whole flank in males (vs. absence); uniquely in *L. sanguineus*, the bars are broad, wider than the interspace width (vs. narrow, half interspace width or less) and have sinuous margins (vs. straight). *Leptopanchax sanguineus* is further distinguished from *L. splendens* by having 15 dorsal-fin rays (vs. 12–14), 6 pelvic-fin rays (vs. 5), 27 scales on the longitudinal series and 9 on the transverse series (vs. 24–25 and 7, respectively), 29 vertebrae (vs. 26–27), pelvic fin tip posteriorly reaching the anal fin in males (vs. reaching urogenital papilla), pelvic-fin bases medially separated, in close proximity (vs. medially united), absence of filamentous rays on the caudal fin (vs. short filamentous rays on the posterior margin of the caudal fin in males), presence of a golden stripe on the distal margin of the dorsal fin in males (vs. white stripe), absence of contact organs on the male pectoral fin (vs. presence) and absence of the dermosphenotic bone (vs. presence). *Leptopanchax sanguineus* also differs from *L. splendens* and all other cynopoecilines by the presence of a small red spot on the posterior portion of the iris (vs. spot absent).

#### Description.

Morphometric data appear in Table 1. Body slender, sub-cylindrical. Greatest body depth at vertical just anterior to pelvic-fin base. Dorsal and ventral profiles of head and trunk slightly convex, approximately straight on caudal peduncle. Head narrow, subtriangular in lateral view. Jaws short, teeth numerous, conical, irregularly arranged; outer teeth hypertrophied, inner teeth small and numerous. Vomerine teeth absent. Urogenital papilla cylindrical and short in males, slightly projecting body-wall outside, and pocket-shaped in females.

Dorsal fin subtriangular, pointed and terminating in short filamentous ray in males, its tip posteriorly reaching vertical through caudal-fin base; dorsal fin slightly pointed to rounded in females. Anal fin sub-rectangular, pointed and longer posteriorly in males, rounded in females. Caudal fin elliptical to sub-lanceolate in males, slightly longer than deep, often posteriorly terminating in minute tip; caudal fin elliptical in females. Pectoral fin elliptical, posterior margin reaching between base of pelvic-fin base and anus. Pelvic fin small, tip reaching anal-fin origin; pelvic-fin bases separated, medially in close proximity. Dorsal-fin origin at vertical between base of 4^th^ and 5^th^ anal-fin rays. Dorsal-fin rays 15; anal-fin rays 18; caudal-fin rays 28; pectoral-fin rays 15; pelvic-fin rays 6. No contact organs on fins. Four neuromasts on caudal-fin base. Total vertebrae 29.

Scales small, cycloid. Body and head entirely scaled, except anterior ventral surface of head. Body squamation extending over anterior 20 % of caudal-fin base; no scales on dorsal, anal and pectoral-fin bases. Frontal squamation E-patterned; E-scales not overlapping medially; supraorbital scales absent. Longitudinal series of scales 27; transverse series of scales 9; scale rows around caudal peduncle 12. Three, or four, minute contact organ per scale of ventral portion of flank in males. Cephalic neuromasts: supraorbital 1 + 10; parietal 1; anterior rostral 1, posterior rostral 1; infraorbital 1 + 15; preorbital 3; otic 1, post-otic 2; supratemporal 1; median opercular 1, ventral opercular 1; pre-opercular 11, mandibular 7; lateral mandibular 4, paramandibular 1.

#### Colouration in life.

***Males***. Flank light metallic blue with 12 or 13 red bars, wider than interspace, margins sinuous producing overall zigzag shape. Dorsum pale yellowish brown, venter pale blue with red bars. Head light blue to greenish blue on opercle, with red reticulation; red stripe between orbit and middle opercle. Jaws red. Ventral surface of head pale blue, scale margin red. Iris bright blue, with dark reddish-brown bar through orbit centre, and small red spot on its posterior margin. Unpaired fins red with metallic blue to greenish-blue vertical vermiculate marks; broad golden stripe on distal margin of dorsal fin. Pelvic fin red with bright blue margin. Pectoral fin hyaline.

***Females*.** Flank pale brownish grey. Dorsum pale brown, venter white. Head side grey, with pale golden iridescence on opercle. Iris yellow, with dark brownish grey bar through orbit centre, and small red spot on its posterior margin. Fins hyaline.

#### Colouration in alcohol.

In both sexes, specimens with head and flank pale brown; fins hyaline in females, hyaline with pale brown pigmentation in males.

#### Distribution and habitat.

**Figure 3.**
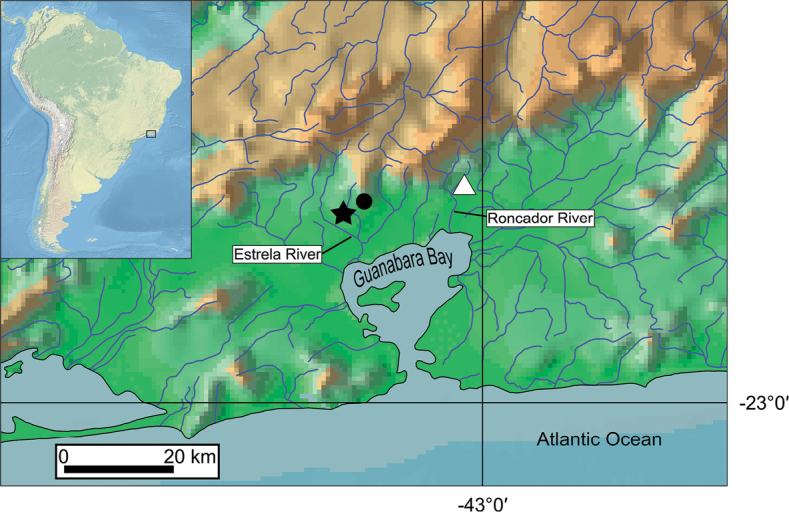
Geographical distribution of *L. sanguineus* sp. nov. (white triangle) and *L. splendens* (black symbols: star, type locality; dot, 2018 collection site).

**Figure 4.**
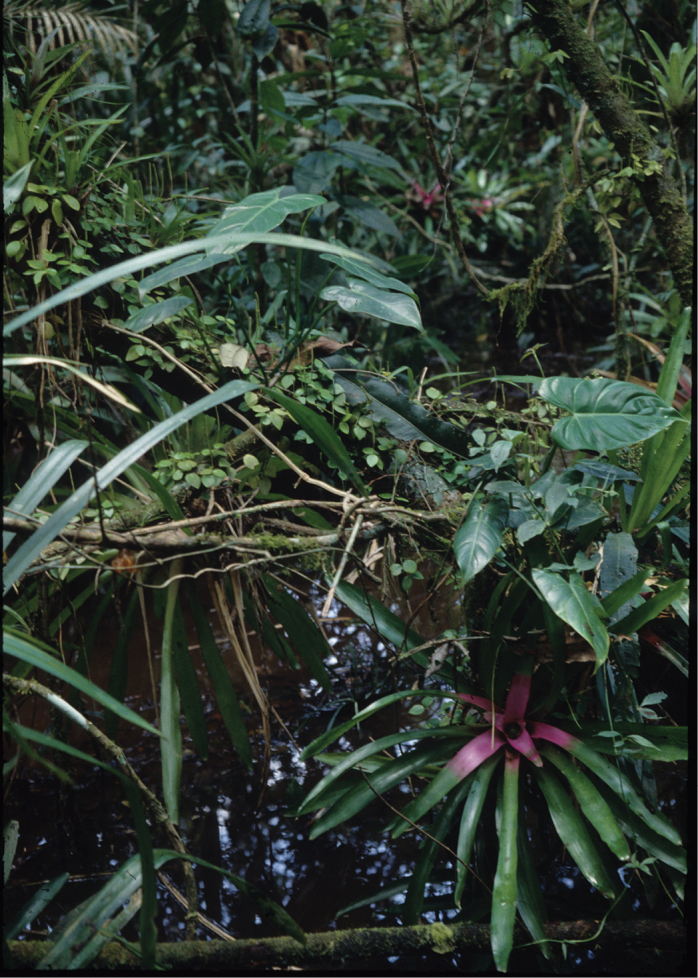
Habitat of *L. sanguineus* sp. nov. in 1988.

*Leptopanchax sanguineus* is known from specimens collected between 1985 and 1987, from a single locality (Fig. 3). The collection site was situated in a dense moist forest, consisting of a well-preserved fragment of about 200,000 m^2^, of the original forest that formerly occupied the plains surrounding the coastal mountain range. This forest is a campsite used by a group of Boy Scouts (Campo Escoteiro Geraldo Hugo Nunes). It is drained by small streams belonging to the Magé River Basin (also known as Roncador River Basin). *Leptopanchax sanguineus* was found in temporary swamp channels situated in small depressions but not directly in contact with surrounding streams. These channels were shallow, about 20 cm deep, with clear, slightly yellow water, and no aquatic vegetation (Fig. 4) (see also Costa 1995: figs 128, 129). The water was acid, pH usually 4.8 to 6.0 after rains (Tanizaki et al. 1991), and the bottom composed of dense litter.

In 1988, some killifish breeders tried to breed *L. sanguineus* in aquaria, but offspring contained only male specimens (J. C. Ghisolfi pers. comm. 1990). Between 1989 and 2000, annual attempts were made to collect the species again. Using GPS, the exact point of the original collection was recorded and new sites were sampled, but no specimen was found. In 2001, monthly collections were made but again no specimen of *L. sanguineus* was found. The shallow temporary swamp channels disappeared, probably as a result of the lowering of the water table caused by the diversion of waters from the streams to supply an ornamental fish farm in the vicinity of the forest (Costa 2009). Sporadic attempts to find this species in other localities of the Magé River Basin, including its upper and lower courses were also unsuccessful. These attempts were directed for all areas with suitable environmental conditions for seasonal killifishes inhabiting moist forests (i.e., flooded forested plains). Since 2009, the Campo Escoteiro Geraldo Hugo Nunes became officially protected by the Brazilian Government when it was recognised as a private natural heritage reserve. However, *L. sanguineus* has not been found since 1987 and it is possibly extinct in the area.

#### Etymology.

The name *sanguineus*, from the Latin, meaning blood-coloured, is an allusion to the predominantly red colouration in males, unique among Neotropical killifishes.

## Discussion

Morphological data of *L. sanguineus*, then identified as *L. splendens*, were used in a phylogenetic analysis of the Cynopoecilini (Costa 2016a), justifying its inclusion in the genus *Leptopanchax* (Costa 2016b). *Leptopanchax sanguineus* shares with other congeners a golden distal stripe on the dorsal fin in males and vermiculate marks on the caudal fin in males (Fig. 2A). These apomorphic character states are present in all species placed by Costa (2016b) in *Leptopanchax*, including *L. citrinipinnis*, the type species of the genus (Fig. 2C–F), although the golden stripe on the dorsal fin is not always well delimited and vermiculate marks on the caudal fin may acquire different shapes. A third synapomorphy described by Costa (2016b) to diagnose *Leptopanchax* is the presence of an elliptical caudal in males, longer than deep (Fig. 2C–F), a condition that is not clearly attributed to *L. sanguineus*, in which the caudal fin is shorter and its posterior margin is slightly pointed (Fig. 2A). Among species of *Leptopanchax*, *L. sanguineus* was considered by Costa (2016b) to be closer to *L. aureoguttatus* (Cruz, 1974) and *L. itanhaensis* (Costa, 2008), which also have a horizontal red stripe between the orbit and the preopercle in males (Fig. 2A, C, D). *Leptopanchax sanguineus* differs from all other cynopoecilines by having red bars on the male flank that are wider than the interspace and have sinuous margins, and the presence of a small red spot on the posterior portion of the iris (Fig. 2A).

The recent rediscovery of *L. splendens* poses some incongruence in its positioning. This species also has a red stripe between the orbit and the preopercle and shares with *L. sanguineus* the presence of red bars on the whole flank and blue iris in males (Fig. 2B), but it does not have the character states diagnostic for *Leptopanchax*. In *L. splendens*, there is no broad golden distal stripe on the male dorsal, but a narrow bluish white stripe, and there are no vermiculate marks on the male caudal fin, which is pale orange, with a few pale red bars on the basal region (Fig. 2B). On the other hand, the series of narrow straight red bars on the male flank in *L. splendens* (Fig. 2B) may be non-homologous to the irregular broad red bars in *L. sanguineus* (Fig. 2A), but similar to the narrow straight bars present on the anterior portion of the male flank in *Mucurilebias leitaoi* (Cruz & Peixoto, 1991), which also exhibits a narrow white stripe on the dorsal fin (Cruz and Peixoto 1991; Costa 2014). Therefore, based on available data, the phylogenetic position of *L. splendens* remains unclear, and possibly it is not closely related to *L. sanguineus*, although both species are endemic to neighbouring river basins (Fig. 3). The uncertain phylogenetic position of *L. splendens* is also reinforced by the presence of a dermosphenotic bone and male contact organs on the male pectoral fin, which are plesiomorphic conditions for cynopoecilines. These two plesiomorphic character states occur in species of the genera *Mucurilebias* Costa, 2014 and *Notholebias* Costa, 2008, but not in species of the clade comprising *Leptopanchax*, *Campellolebias* Vaz-Ferreira & Sierra, 1974, *Cynopoecilus* Regan, 1912, and *Leptolebias* Myers, 1952, in which the dermosphenotic and pectoral-fin contact organs are always absent (Costa 2016a).

*Leptopanchax sanguineus*, known from a single locality, is a typical moist-forest species, an ecological adaptation considered to have arisen three times independently among cynopoecilines (Costa 2016a). Presently, only six cynopoeciline species are adapted to live in this kind of habitat: *Cynopoecilus notabilis* Ferrer, Wingert & Malabarba, 2014, *Leptolebias marmoratus*, *Leptop. aureoguttatus*, *Leptop. itanhaensis*, *Leptop. sanguineus*, and *Leptop. splendens*. Records of cynopoeciline forest-dwellers are extremely rare when compared with records of cynopoeciline species found in open vegetation habitats. For example, in southern Brazil, species of *Cynopoecilus*, subgenus Cynopoecilus, that are always found in open vegetation habitats, are frequently sampled in field studies (Costa 2002c; Costa et al. 2016). Contrastingly, the only species of the subgenus Poecilopanchax Costa, 2016, *C. notabilis*, is known from a single locality (Ferrer et al. 2014). Similarly in south-eastern Brazil, where a rich species diversity of cynopoecilines is concentrated (Costa 2009), species of the genus *Notholebias*, which are found in open vegetation habitats, are known from several localities (Costa 1988) and are often recorded in the literature (e.g. Costa and Amorim 2013), whereas each of the three species endemic to the BGA adapted to life within dense forests, *Leptol. marmoratus*, *Leptop. sanguineus* and *Leptop. splendens*, were recorded a single time in the last 30 years of continuous field studies directed to killifish habitats in the region (Costa and Lacerda 1988; Costa 2002c; present study). Actually, this discrepancy in field records is associated with the intense deforestation process in the Atlantic Forest (Costa 2008). Several reserves have protected moist and semi-deciduous forests along the coastal mountain range (Serra do Mar), but lowland moist forests, where killifishes are found, were greatly extirpated during the last two centuries and are presently represented by rare small fragments. Therefore, killifish species uniquely inhabiting lowland moist forests should be regarded with special concern in evaluation studies for conservation priorities.

### Key to identification of *Leptopanchax* species

**Table:** 

1	Flank in males with continuous longitudinal rows of iridescent light blue to yellowish green spots on each scale (Fig. 2E–F); no horizontal red stripe on head side (Fig. 2E–F); dorsal-fin origin in vertical between base of seventh and ninth anal-fin rays	**2**
–	Flank in males with interrupted zones of iridescent marks (Fig. 2A–D); horizontal red stripe between orbit and preopercle in males (Fig. 2A–D); dorsal-fin origin in vertical between base of second and sixth anal-fin rays	**3**
2	Well-delimited dark-red stripe on distal margins of dorsal and anal fins in males (Fig. 2F)	*** L. opalescens ***
–	Diffuse dark-reddish brown pigmentation on distal margins of dorsal and anal fins in males (Fig. 2E)	*** L. citrinipinnis ***
3	Iris bright greenish yellow in males (Fig. 2C, D); no bars on flank (Fig. 2C, D)	**4**
–	Iris bright blue in males (Fig. 2A, B); red bars on flank in males (Fig. 2A, B)	**5**
4	Two dark-red stripes along entire dorsal and ventral submarginal parts of caudal fin in males (Fig. 2C); male caudal-fin stripes branching posteriorly (Fig. 2C); dark red to dark brown short transverse bars on basal portion of dorsal fin in males (Fig. 2C)	*** L. aureoguttatus ***
–	Two dark-red stripes on anterior portion of dorsal and ventral submarginal parts of caudal fin in males (Fig. 2D); male caudal-fin stripes not branching posteriorly (Fig. 2D); small brownish orange spots on basal portion of dorsal fin in males (Fig. 2D)	*** L. itanhaensis ***
5	Flank in males with narrow red bars, narrower than interspace width, with straight margins (Fig. 2B); distal margin of dorsal fin with narrow bluish white stripe in males (Fig. 2B); 12–14 dorsal-fin rays; 5 pelvic-fin rays; 24–25 scales on longitudinal series; 7 scales on transverse series; pelvic fin tip posteriorly reaching urogenital papilla; pelvic-fin bases medially united; with filamentous rays on distal margin of dorsal fin and posterior margin of caudal fin in males (Fig. 2B)	*** L. splendens ***
–	Flank in males with broad red bars, wider than interspace width, with sinuous margins (Fig. 2A); distal margin of dorsal fin with broad golden stripe in males (Fig. 2A); 15 dorsal-fin rays; 6 pelvic-fin rays; 27 scales on longitudinal series; 9 scales on transverse series; pelvic fin tip posteriorly reaching anal fin in males; pelvic-fin bases medially separated; absence of filamentous rays on fins (Fig. 2A)	***L. sanguineus* sp. nov.**

## Supplementary Material

XML Treatment for Leptopanchax sanguineus
